# Expression of Pinopodes in the Endometrium from Recurrent Pregnancy Loss Women. Role of Thrombomodulin and Ezrin

**DOI:** 10.3390/jcm9082634

**Published:** 2020-08-13

**Authors:** Silvia D’Ippolito, Fiorella Di Nicuolo, Massimiliano Papi, Roberta Castellani, Valentina Palmieri, Valeria Masciullo, Vincenzo Arena, Chiara Tersigni, Micaela Bernabei, Alfredo Pontecorvi, Giovanni Scambia, Nicoletta Di Simone

**Affiliations:** 1 Dipartimento di Scienze della Salute della Donna, del Bambino e di Sanità Pubblica, Fondazione Policlinico Universitario A. Gemelli, Istituto di Ricovero e Cura a Carattere Scientifico (I.R.C.C.S.), 00168 Roma, Italy; valeria.masciullo@policlinicogemelli.it (V.M.); vincenzo.arena@policlinicogemelli.it (V.A.); chiara.tersigni@policlinicogemelli.it (C.T.); giovanni.scambia@policlinicogemelli.it (G.S.); 2 Paolo VI International Scientific Institute, Università Cattolica del Sacro Cuore, 00168 Roma, Italy; fiorella.dinicuolo@gmail.com (F.D.N.); alfredo.pontecorvi@policlinicogemelli.it (A.P.); 3 Dipartimento di Neuroscienze, Università Cattolica del Sacro Cuore, Fondazione Policlinico Universitario A. Gemelli Istituto di Ricovero e Cura a Carattere Scientifico (I.R.C.C.S.), 00168 Roma, Italy; massimiliano.papi@unicatt.it (M.P.); valentina.palmieri@unicatt.it (V.P.); 4 Dipartimento di Scienze della Vita e Sanità Pubblica, Università Cattolica del Sacro Cuore, 00168 Roma, Italy; roberta.castellani@unicatt.it; 5 Istituto di Anatomia e Istologia Patologica, Università Cattolica del Sacro Cuore, 00168 Roma, Italy; micaela.bernabei@unicatt.it; 6 Dipartimento di Scienze Gastroenterologiche, Endocrino-Metaboliche e Nefro-Urologiche, Fondazione Policlinico Universitario A. Gemelli, Istituto di Ricovero e Cura a Carattere Scientifico (I.R.C.C.S.), 00168 Roma, Italy; 7 Istituto di Patologia Medica, Università Cattolica del Sacro Cuore, 00168 Roma, Italy

**Keywords:** recurrent pregnancy loss, endometrium receptivity, thrombomodulin, ezrin, cytoskeleton, cytoskeletal actin filaments, personalized medicine

## Abstract

Background: Pinopode expression has been suggested as a marker of endometrial receptivity. Methods: We set up an experimental study comparing endometrial tissue from recurrent pregnancy loss (RPL, *n* = 30) and fertile control (CTR, *n* = 20) women in terms of pinopode expression/morphology; expression of thrombomodulin (TM) and ezrin; cytoskeletal organization. Endometrial samples were collected during implantation window and evaluated by scanning electron microscopy, western blot, and immunofluorescence. Results: We found that RPL endometrial tissue showed: (i) increased pinopodes density (* *p* < 0.05); (ii) a reduced diameter of pinopodes (* *p* < 0.05); (iii) a decreased TM and ezrin expression (*p* < 0.05). Additionally, confocal images showed a significantly reduced expression of phosphorylated (*p*)-ezrin, confirming the results obtained through immunoblot analysis. Immunofluorescence staining showed that in CTR samples, junctions between cells are intact and clearly visible, whereas actin filaments appear completely lost in RPL endometrial samples; this suggests that, due to the impaired expression and activity of TM and ezrin, actin does not bind to plasma membrane in order to orchestrate the cytoskeletal actin filaments. Conclusions: Our findings suggest that an impaired expression of TM and expression/activation of ezrin may affect the connection between the TM and actin cytoskeleton, impairing the organization of cytoskeleton and, eventually, the adequate pinopode development.

## 1. Introduction

Recurrent pregnancy loss (RPL), defined as the occurrence of two or more consecutive pregnancy losses [1], recognizes as common established causes uterine anomalies, thrombophilic, hormonal and metabolic disorders, cytogenetic and sperm abnormalities [2]. In spite of extensive research on this obstetric condition, almost half of the cases of RPL remain unexplained and many efforts are being made, both to improve the knowledge in this field and to shorten the time needed to achieve a successful pregnancy. It has been suggested that RPL may result from an impaired endometrial receptivity during the implantation window [3]. This period of receptivity is short and results from the programmed sequence of the action of estrogens and progesterone on the endometrial cells, which undergo specific changes to promote the implantation of the free-lying blastocyst. In particular, endometrial stromal cells, once decidualized, produce critical signals that act on the endometrial endothelial cells and promote their proliferation and differentiation [4,5,6]. Furthermore, the luminal surface of endometrial cells undergoes ultrastructural changes, known as pinopodes, which arise from the apical membrane like balloon-like projections, measuring several micrometers in diameter. The development/maturation of pinopodes, expressed along with filamentous actin under the apical plasma membrane surface, is strictly regulated by the actin filaments of cytoskeleton [7,8,9]. Their function is not well known. They have been associated to molecular markers of endometrial receptivity, like integrins, leukemia inhibiting factor (LIF), l-selectin, glutaredoxin, glycodelin-A, microRNAs and, consistently: (i) their development coincides with the implantation window; (ii) the attachment of a blastocyst occurs at the site of pinopode expression *in vitro*; (iii) the pinopode surface shows receptors for adhesion molecules that are fundamental for embryo attachment to the uterine mucosa [10,11,12,13]. In conclusion, the ultrastructural organization of endometrial cells and the expression of pinopode represent an important part of the complex process of implantation and are crucial steps for adequate placentation at the beginning of pregnancy.

During the last few years, scientists have reported the important role on placentation played by an integral membrane protein, known as thrombomodulin (TM) [14,15]. This protein belongs to the protein C system, which acts as a strong anticoagulant factor, and is expressed on the endothelial surface of blood vessels, mainly in the microvasculature, keratinocytes, intestinal epithelial cells, trophoblast cells and, as shown, on endometrial stromal cells [15,16,17,18,19]. Previous studies reported that: (i) the deletion of placental TM induces embryonic death at day 8.5 post-coitum in murine models [15,20]; (ii) the selective reconstitution of TM in trophoblast of the early placenta is able to prevent the early embryonic lethality [15]; and (iii) placentas obtained from patients with spontaneous recurrent miscarriage show a significantly reduced expression of TM [21,22,23]. Of note, miscarriage does not seem attributable to the uncontrolled procoagulant state, since histopathological findings specific of thrombosis have not been detected in the majority of miscarriage samples [21]. Further recent studies report a loss or a reduction in TM expression in placentas from preeclamptic women, hence confirming the important role of this protein during early placentation [20,21,22,23]. Even though several observations suggest the important role of TM during pregnancy, the mechanisms by which this protein contributes to placental development remain unclear. Beyond anticoagulation, TM inhibits inflammatory pathways, participates in cell-cell adhesion, and interacts with the actin filaments of cytoskeleton, enhancing cellular barrier integrity. The link between TM and the actin filaments is mediated by ezrin, an important bridge protein existing either in the unphosphorylated-inactive or in the phosphorylated-active conformation [19,24,25].

To date, no studies have been performed on endometrial TM and its possible role on the endometrial receptivity and in particular on endometrial pinopodes. Given the ability of TM-ezrin complex to interact with the actin filaments of cytoskeleton and the crucial role played by actin filaments of cytoskeleton in the expression and organization of pinopodes, the aim of our research is to investigate whether a correlation exists between the pinopode expression and morphology and the endometrial expression of both these proteins. In this direction, we intend to compare the expression of pinopodes, TM, and ezrin (total and phosphorylated form) in the endometrium from RPL and fertile women. In addition, we aim at investigating the actin filaments of cytoskeleton organization in endometrial tissue from both groups of women. Our findings help achieve improved precision in the evaluation of endometrial receptivity that could enable a better synchronization of the embryo and the endometrium prior to implantation. In addition, this manuscript improves the current understanding of embryo attachment to endometrial tissue and, based on a model of precision medicine, might represent a useful tool, possibly commercially available, able to define either a receptive or an unreceptive endometrium.

## 2. Experimental Section

### 2.1. Methods

All methods were carried out in accordance with relevant guidelines and regulations.

#### 2.1.1. Patients

This study was performed from January to December 2019 at the Recurrent Pregnancy Loss Unit, Dipartimento di Scienze della Salute della Donna, del Bambino e di Sanità Pubblica, Fondazione Policlinico Universitario A. Gemelli IRCCS, Università Cattolica del Sacro Cuore, Rome, Italy.

The study population included women with ≥2 previous uncomplicated term pregnancies (control group, CTR) and women with history of idiopathic RPL with ≥3 spontaneous pregnancy losses (≤12 weeks of gestation) clinically documented by ultrasonography and histopathology examination. The inclusion criteria for both groups were as follows: Caucasian, age ≤39 years, healthy, regular ovulatory cycles (28–32 days), normal endocrine profile, normal serum levels of follicle-stimulating hormone (FSH < 10 mIU/mL), luteinizing hormone (LH< 10 mIU/mL) and anti-mullerian hormone (AMH > 2 ng/mL) on day 3 of the menstrual cycle, absence of abnormal ovarian and endometrial ultrasonographic features, no use of any contraceptive drugs or intrauterine device in the last six months.

Exclusion criteria were as follows: presence of abnormalities at the screening for RPL (anatomical abnormalities-severe hydrosalpinx, advanced endometriosis (stage III–IV), uterine adhesion, or myoma or adenomyosis adjacent to uterine cavity; luteal phase deficiency; hyperprolactinemia; hyperinsulinemia or insulin resistance; endocrine disorders; vaginal and/or endometrial infections; karyotype anomalies; thrombophilic disorders; and autoimmune diseases), smoking, obesity, and alcohol consumption. All women gave their informed consent to use, anonymously, their data for research purposes, and the protocol was approved by the ethics committee of the A. Gemelli Universitary Hospital, Università Cattolica del Sacro Cuore, Rome, Italy (protocol number 36401/16 ID:1355). The women were advised to avoid pregnancy in the month in which hysteroscopy was carried out.

#### 2.1.2. Endometrial Samples

Women underwent hysteroscopic biopsy during the putative window of implantation. The timing of the biopsy was dated according to the LH surge (mid luteal phase timed on the seventh day after LH surge, LH + 7) and was confirmed by histologic assessment [26]. Serum progesterone and β-hCG levels were determined in the day of biopsy. Endometrial biopsies were performed using a 3-mm Novak curette for cultural and for functional research purposes. Extreme care was taken during endometrial sampling to avoid any contact between the curette and the vaginal walls. With regard to infectious agents, common bacteria like Chlamydia trachomatis, Mycoplasma, Ureaplasma urealyticum, Neisseria gonorrheae, and yeasts were looked for at endometrial levels. In the case an infectious agent was detected, an appropriate treatment was prescribed. The endometrial biopsies with infections were not included in the study. Patients with evidence of chronic endometritis at hysteroscopy (about 20%) were not included in the study. The samples were washed immediately in normal saline and stored at −80 °C.

#### 2.1.3. Scanning Electron Microscopy

Pinopode expression and morphology were evaluated by Scanning Electron Microscopy (SEM) (Zeiss, Dresden, Germany) after fixation of samples, as reported previously [27]. Briefly, samples were fixed in 4% paraformaldehyde, dehydrated in a series of ethanol solutions, and sputter coated. Images were acquired with an SEM Supra 25 (Zeiss, Dresden, Germany) with a secondary electron detector. Image analysis was performed by FIJI software [28]. The number of pinopodes per area was manually counted and the diameter of pinopodes was measured with FIJI software.

#### 2.1.4. Western Immunoblotting

Thrombomodulin and ezrin (total and phosphorylated conformation) expression was analyzed by western immunoblotting. Total lysates obtained from endometrial biopsies (RIPA buffer:150 mM NaCl, 50 mM Tris, pH 8.0, 0.1% SDS, 0.25% deoxycholate, 1% NP-40, with protease inhibitor cocktail), were separated by 10% SDS-PAGE electrophoresis under reducing conditions. After gel electrophoresis, proteins were transferred to a PVDF membranes (Millipore). The membranes were blocked at room temperature for 1 h in 5% non-fat dry milk, and incubated overnight at +4 °C with a specific primary antibody (anti-TM, ThermoFisher Scientific, Rockford, IL, USA; anti-total and -phosphorylated ezrin (pTyr353); Cell Signaling Technology, Danvers, MA, USA.). The membranes were washed with PBST and incubated in specific horseradish peroxidase-conjugated IgG diluted 1:2000 in 5% non-fat dried milk in PBST. Bound secondary antibody was detected by chemiluminescence. Bands were analyzed using the enhanced chemiluminescence (ECL™ Amersham, UK) by chemiluminescence imaging system, Alliance 2.7 (UVITEC, Cambridge, UK) and quantified by the software Alliance V_1607. The levels of TM and ezrin (total and phoshorilated) were estimated versus the constant level of β-tubulin (anti-β-tubulin antibody, Sigma-Aldrich, St Louis, MO, USA).

#### 2.1.5. Immunofluorescence Staining

The endometrial cytoskeletal organization was studied by immunofluorescence. Endometrial tissue sections were prepared by quick freezing in optimal cutting temperature (OCT) medium. Frozen samples were sectioned using a cryostat and allowed to air dry on the slide 10–15 min prior to fixation. Sections were fixed with 3% paraformaldehyde/phosphate-buffered saline, and permeabilized with 0.2% Triton-X 100. After blocking with 0.2% bovine serum albumin for 30 min, cells were incubated with anti-phosphorylated ezrin antibody (Cell Signaling Technology, Inc., Leiden, The Netherlands) at 1:100 dilution for 30–60 min, followed by rhodamine-labeled secondary antibody for 60 min (Molecular Probe, Eugene, OR, USA) and, after washing with phosphate-buffered saline, incubated with FITC-phalloidin (Invitrogen/Life Technologies, Waltham, MA, USA) at 1:200 dilution for 20 min. Stained endometrial tissues were mounted with VectaShield (Vector Laboratories, Burlingame, CA, USA) mounting medium and visualized using a confocal microscope (Nikon A1 MP, Nikon Instruments Europe BV, Amsterdam, The Netherlands) equipped with an on-stage incubator. Images were obtained exciting at 402 nm and revealing 450 ± 25 nm at for DAPI, while FITC-phalloidin was excited at 488 nm and emission was recorded at 525 nm ± 50 nm. Rhodamine signal was imaged exciting samples with 561 nm and recording emission at 595 ± 25 nm. Image analysis was performed using FIJI software.

#### 2.1.6. Statistical Analysis and Data Availability

The results are presented as the mean ± standard error (SE). The data were analysed using one-way analysis of variance (ANOVA) followed by a post-hoc test (Bonferroni test). Statistical significance was determined at *p* < 0.05. The data that support the findings of this study are available on request from the corresponding author.

## 3. Results

### 3.1. Patients

The study population included 30 RPL and 20 CTR women. The clinical characteristics of the patients are outlined in Table 1. The RPL and CTR groups were similar with respect to age and body mass index (BMI). Of the included women, 15 RPL and 10 CTR biopsies were used for scanning electron microscopy analysis; 8 RPL and 6 CTR biopsies for the immunoblot analysis; and 7 RPL and 4 CTR biopsies for immunofluorescence staining. 

### 3.2. Scanning Electron Microscopy

**Figure 1:**
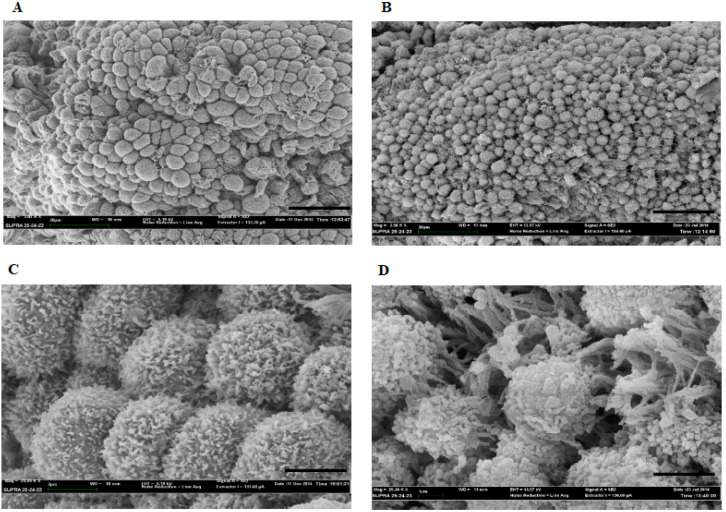
Expression of pinopodes in endometrial tissue from control and recurrent pregnancy loss women. Representative Scanning Electron Microscopy (SEM) pictures of the apical surface of the endometrial tissue from control (CTR; (**A**,**C**)) and recurrent pregnancy loss (RPL; (**B**,**D**)) women, during the window of implantation. Pinopodes can be seen extending from the surface of luminal endometrial tissue. Scale bar is 20 µm (**A**,**B**) and 2 µm (**C**,**D**). CTR: control women; RPL: recurrent pregnancy loss women.

**Figure 2:**
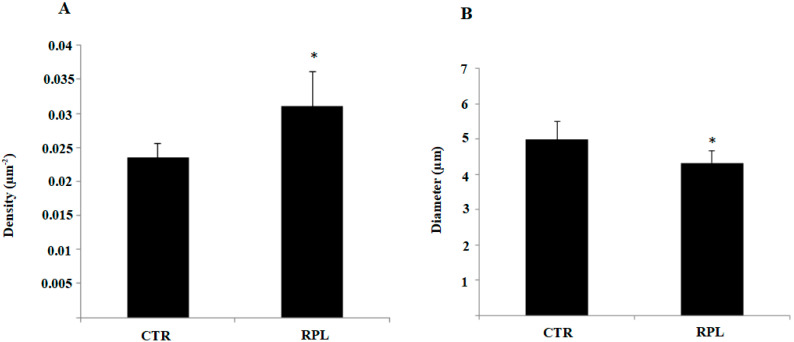
Quantitative Analysis of pinopode expression in the endometrium from control and recurrent pregnancy loss women. (**A**) Scanning Electron Microscopy (SEM) image analysis allowed to calculate the mean density and diameter of pinopodes. The quantification of SEM image analysis demonstrated an increase of pinopode density in RPL endometrium as compared to control tissues (* *p* < 0.05). (**B**) When considering the morphology, pinopodes from RPL women showed a statistically significant reduced diameter (* *p* < 0.05). Values are means ± SD of 10 covered areas; data are representative of 30 RPL and 20 CTR women. CTR: control women; RPL: recurrent pregnancy loss women.

SEM was used to explore the pinopode number and morphology in the endometrium from CTR and RPL women. Figure 1 shows representative SEM pictures of endometrial tissue from CTR (Figure 1A,C) and RPL (Figure 1B,D) women during the window of implantation at different magnifications. In particular, scale bar is 20 µm (Figure 1A,B) and 2 µm (Figure 1C,D). SEM image analysis allowed calculating the mean number per area (expressed as density, µm^−2^) and the diameter of pinopodes. The analysis demonstrated an increase of pinopodes density in RPL endometrial tissues as compared to CTR (Figure 2A, * *p* < 0.05). When considering the morphology, a significantly reduced diameter was found in pinopodes from the RPL endometrium (Figure 2B, * *p* < 0.05), hence suggesting a lack of full completeness in the process of pinopode development/maturation and, therefore, an alteration in endometrial receptivity. Values are means ± SD of ten covered areas; data are representative of biopsies from 15 RPL and 10 CTR women. Data are representative of ten independent experiments.

### 3.3. Immunoblot Analysis

**Figure 3:**
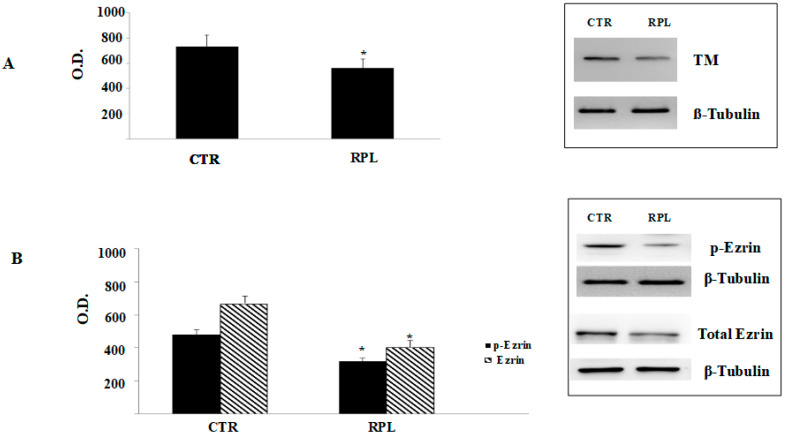
Thrombomodulin and ezrin expression in endometrial tissue from control and recurrent pregnancy loss women. (**A**) Western immunoblot analysis of thrombomodulin (TM) expression in lysates of endometrial biopsies from CTR and RPL women during the putative window of implantation, timed to the LH surge. Significantly decreased levels of TM in RPL as compared to CTR lysates were found (* *p* < 0.05). (**B**) Consistent with these findings, in parallel experiments, a statistically significant reduction of both the phosphorylated and the total levels of ezrin in samples from RPL women it was observed, as compared with CTR (* *p* < 0.05). Proteic bands are representative images cropped from the full length gels obtained from RPL and CTR women, included in Figure S1 (for TM) and Figure S2 (for total and *p*-ezrin). ß-Tubulin is shown as a loading control. Data are representative of three independent experiments. TM: thrombomodulin; CTR: control women; RPL: recurrent pregnancy loss women.

Phosphorylation of ezrin at tyrosine 353 (*p*-ezrin) is required to activate ezrin from the inactive conformation, hence favouring the subsequent cytoskeletal rearrangements that are involved in cell-cell attachments and membrane specializations [8,25]. Once activated, ezrin interacts with the extracellular lectin-like domain of TM in order to control cell morphology [25]. Western immunoblot analysis in endometrial biopsies, obtained during the putative window of implantation, timed to the Luteal Hormone (LH) surge (mid luteal phase timed on the 7th day after LH surge, LH  +  7), revealed a significantly decreased levels of TM in lysates obtained from RPL women compared to controls (*p* < 0.05; Figure 3A). In parallel experiments, we also observed a significant reduction of both the phosphorylated and total levels of ezrin in samples from RPL patients, as compared with the controls (*p* < 0.05; Figure 3B). Proteic bands are representative images cropped from the full length gels obtained from RPL and CTR women, included in Supplementary Figure S1 (for TM) and Supplementary Figure S2 (total and *p*-ezrin). Data are representative of three independent experiments (8 RPL and 6 CTR biopsies) and strongly suggest that the impaired TM/ezrin expression and activation in RPL endometrium are parallel to the differences observed in the pinopode development between the two groups.

### 3.4. Co-Localization of Ezrin and Actin Filaments of Cytoskeleton

**Figure 4:**
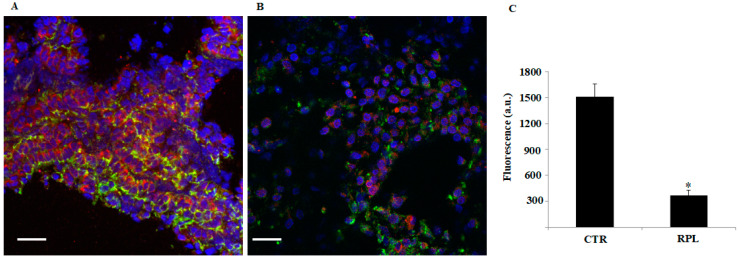
Co-localization of ezrin and actin filaments of cytoskeleton in the endometrial tissue from control and recurrent pregnancy loss women. (**A**) Immunofluorescence staining of endometrial tissue from CTR women. Scale bar is 20 µm (**B**) Immunofluorescence staining of endometrial tissue from RPL women. Endometrial tissue was fixed, permeabilized, and doubly stained with anti-phosphorylated ezrin antibody (rhodamine, red) and FITC-phalloidin (green) antibodies and analyzed by confocal fluorescence microscopy. Nucleus staining was performed with DAPI. Images clearly show a decreased expression of ezrin. Scale bar is 20 µm. (**C**) Quantitative analysis of the images obtained from confocal microscopy (Nikon A1 MP) with the FIJI software. Fluorescence levels were calculated in CTR and RPL tissue. The quantification of fluorescence signal from confocal images showed a significantly reduced expression of ezrin in RPL tissue as compared with control, with a 5-fold decrease of protein expression (* *p* < 0.05). The results are representative of 10 separate experiments. CTR: control women; RPL: recurrent pregnancy loss women.

Ezrin represents the bridge protein connecting the integral membrane protein TM to the intracellular actin filaments cytoskeleton. Immunofluorescence staining of samples was performed to analyze ezrin expression and cytoskeleton organization from 7 RPL and 4 CTR biopsies. Two representative images are reported in Figure 4 with nuclei in blue (DAPI), actin filaments of cytoskeleton in green (Phalloidin), and Ezrin in red (Rhodamine) in control (Figure 4A) and RPL endometrium (Figure 4B), respectively. Quantification of fluorescent signal from confocal images showed a significantly reduced expression of *p*-ezrin, confirming the results obtained through immunoblot analysis, with a 5-fold decrease of protein expression (Figure 4C). Our findings suggest that an impaired expression/activation of ezrin might affect the connection between the TM and actin filaments of cytoskeleton, hence impairing the organization of cytoskeleton and, finally, the correct pinopode development. The results are representative of 10 separate experiments. Our findings suggest that an impaired expression/activation of ezrin might affect the connection between the TM and actin filaments of cytoskeleton, hence impairing the organization of cytoskeleton and, finally, the correct pinopode development.

### 3.5. Immunofluorescence of Actin Filaments of Cytoskeleton

**Figure 5:**
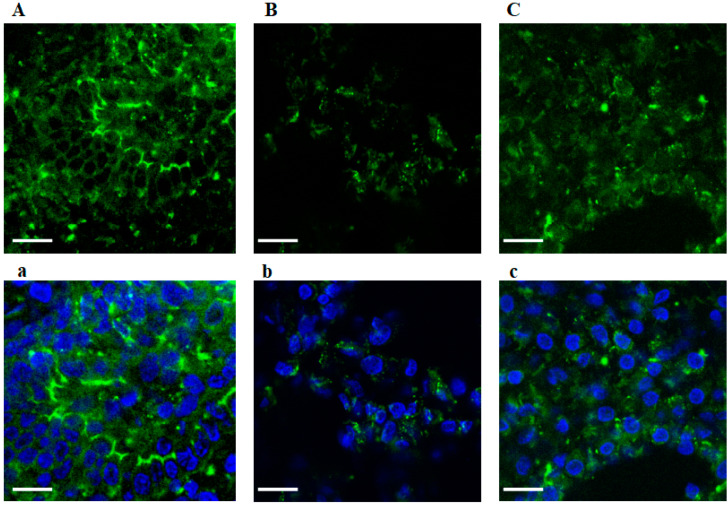
Cytoskeletal actin filaments organization in endometrial tissue from control and recurrent pregnancy loss women. Immunofluorescence staining of cytoskeletal organization in endometrial samples from CTR (Figure **A**,**a**) and RPL (Figure **B**,**b**,**C**,**c**) women. Three representative images are reported in the picture, before and after merging DAPI staining (top and bottom row, respectively). In particular, the top row pictures (Figure **A**,**B**,**C**) show green cytoskeletal staining. The bottom row (Figure **a**,**b**,**c**) show the staining of the nucleus, performed with DAPI. Cytoskeletal organization appears completely lost on RPL samples (green fluorescence, Figure **B**,**b**,**C**,**c**) compared to healthy controls (Figure **A**,**a**), where junctions between cells are intact and clearly visible. Data are representative of 10 independent experiments. Scale bar is 20 µm. CTR: control women; RPL: recurrent pregnancy loss women.

The endometrial cytoskeletal organization was studied by immunofluorescence (shown in Figure 5). The six pictures show immunofluorescence from CTR (Figure 5A,a) and RPL (Figure 5B,b,C,c) endometrial samples. The actin filaments are shown through green fluorescence. The nuclei stained with DAPI are shown in blue (Figure 5a–c). In the CTR samples, junctions between cells are intact and clearly visible. On the contrary, actin filaments appear completely lost in RPL endometrial samples suggesting that, due to an impaired expression and activity of the proteins TM and ezrin, actin is not able to bind to plasma membrane in order to orchestrate the cytoskeletal filaments. Data are representative of 10 independent experiments.

## 4. Discussion

Recurrent pregnancy loss, recently defined as the occurrence of two or more consecutive pregnancy losses, affects approximately 2% to 3% of women and, beyond embryo defects, is associated to endometrial defects and the abnormal expression of various molecules by endometrial cells [1,29]. A correct receptive endometrial state is crucial for fertility as well as placenta formation, since initiation of placentation highly depends on the interaction between trophoblast and endometrium [30]. In the present study, we observed a significant reduction in the diameter of the pinopodes from RPL endometrial tissue. Therefore, the density of pinopodes is higher in RPL tissue as compared to fertile controls. We also found that RPL endometrial tissue shows a significantly reduced expression of both TM and the total and phosphorylated forms of ezrin and that these results are consistent with an altered cytoskeletal organization.

In recent years, the role of the endometrial “maternal side” during early pregnancy is gaining a growing interest and, in particular, the impact of the endometrial receptivity on the pregnancy outcome. Endometrial receptivity refers to a transient period in which ovarian hormones induce changes in endometrial lining in order to allow blastocyst implantation and, in turn, pregnancy initiation [31]. Unfortunately, the “adequate receptive endometrium” is still far from being defined. Several researchers are now attempting to find the most adequate markers of endometrial receptivity; among these are the endometrial pinopodes, projections of the apical cellular surface, which show different stages of development as the luteal phase of the menstrual cycle progresses [32,33,34]. In particular, developing, fully developed, and regressing states have been described, with each phase lasting approximately 24 h. Briefly, developing pinopodes begin to bulge into the uterine lumen, mature pinopodes bulge maximally into the uterine lumen, and regressing pinopodes are slightly wrinkled and less bulging [35,36]. Pinopodes show a variable diameter of 4–6 μm [37,38] according to the state of maturation and their development is closely connected with the dense network of filamentous actin (F-actin), which is expressed under the apical plasma membrane surface [39]. The function of pinopodes is not well known. According to *in vitro* studies, they might represent a site of anchorage for the blastocyst [40]. In addition, they may be involved in the release of secretory vesicles full of Leukemia Inhibitory Factor into the uterine lumen [41]. In humans, pinopodes have been suggested as a reliable marker of the window of implantation [42,43,44]. In a recent randomized controlled trial, authors monitored pinopode expression, analysed the correlation with *in vitro* fertilization (IVF) pregnancy outcomes, and, finally, performed individual embryo transfer response in relation to pinopode scoring. Through the two-year clinical trial, they established a pinopode scoring and found that women with a pinopode scoring cut-off >85 had a higher successful pregnancy rate and required fewer embryo transfer cycles [42].

In our study, by using scanning electron microscopy, we firstly analysed the morphology of endometrial pinopodes. We observed a significantly reduced diameter of pinopodes from RPL women as compared to fertile controls. We may explain our findings with the fact that in the RPL endometrium, pinopodes do not reach a state of complete maturation. Therefore, the density of pinopodes is higher. We suggested this as a possible mechanism useful to prevent any interference to an impaired endometrial receptivity related to the incomplete maturation. The use of pinopode as a marker of endometrial receptivity has important limitations because they show a highly dynamic microstructure and their duration over time is very limited. By taking into account these limitations, in a second step of our research, we considered (i) the strict connection between pinopode expression and actin cytoskeletal organization; (ii) the ability of TM to interact with ezrin to stabilize actin filaments of cytoskeleton; and (iii) the crucial role of TM during early placentation. TM is an integral membrane protein mainly acting as an anticoagulant factor. In addition, the deletion of placental TM induces embryonic death in murine models [15,20] and the selective reconstitution of TM in trophoblast of the early placenta is able to prevent early embryonic lethality [15]. Consistently, a significant reduction of TM expression has been found in placentas obtained from patients with spontaneous recurrent miscarriage [21] and in preeclamptic placentas [22,23], suggesting a crucial role for this protein during early placentation. Since no histopathological thrombotic signs have been found in the majority of the miscarriage/preeclamptic samples, additional properties for TM have been suggested, that is: regulation of apoptotic processes, local control of inflammatory processes, and enhancement to cellular barrier integrity through the stabilization of the actin filaments of cytoskeleton [45,46,47]. To date, no studies have investigated the role of TM on the endometrium. At the time of implantation, estrogens and progesterone induce both the binding of TM to ezrin and the phosphorylation and activation of ezrin. Once activated, ezrin (i) crosslinks F-actin into an orthogonal meshwork, (ii) localizes on the apical pole of cells and (iii) takes part in the actin meshwork observed in the pinopodes [9,25]. It is likely that these cell modifications play a role in embryo adhesion and implantation during the luteal phase. In our research, we investigated whether the different development of pinopodes observed in RPL endometrial tissue may be related to an altered expression of TM and ezrin (total and phosphorylated) and to differences in the organization of the actin filaments of cytoskeleton. Of interest, we found that an altered pinopode development is strictly connected to an altered cytoskeletal organization. We documented, for the first time, that such an alteration is correlated with a down-regulation of TM as well as of ezrin. In this way TM/ezrin may represent an important new indirect marker of impaired pinopode expression and, consequently, of altered endometrial receptivity.

## 5. Conclusions

In conclusion, understanding the mechanisms affecting endometrial receptivity might help to clarify the pathogenic mechanisms underlying defective implantation and placentation. To date, a comprehensive understanding of the impact of endometrium on RPL is still missing. In recent years, much interest has focused on endometrial receptivity with the aim of defining the most adequate state for early implantation. In our study, we demonstrated that a down-regulation of TM-ezrin complex is involved in the alteration of cytoskeletal organization in endometrial cells and that such mechanisms impact pinopode development. Further studies on human endometrium are needed in order to better define the best receptive endometrium. Of interest, we previously demonstrated an increased unfavourable expression of inflammatory cytokines and NACHT-LRR-PYD domains-containing protein 3 (NALP-3) inflammasome in the endometrium from RPL women [48]. In addition, experiments in mouse models of renal interstitial inflammation reported the ability of TM to neutralize the inflammatory response by inhibiting NALP-3 activity [17]. These findings led to hypothesize possible connections between these two complexes. Further studies on human endometrium are needed in order to investigate a possible communication between these two systems at the endometrial level, even in RPL women. The demonstration that a correlation between the two systems exists also at an endometrial level might help in outlining an endometrial set-up unfavorable to the implantation process and therefore in selecting conditions of altered endometrial status.

## Figures and Tables

**Table 1:** Characteristics of women from control and recurrent pregnancy loss (RPL) group.

Characteristics of Women	Control Group (*n* = 20)	Women with RPL (*n* = 30)	*p* Value
Age (years) ± SD	34.7 ± 6.7	37.3 ± 4.3	Ns
BMI (Kg/m^2^)	22.1 (18–30.5)	23.9 (17.6–35.9)	Ns
Smoking status, No (%)	8 (40%)	13 (43%)	Ns
Alcohol consumption (*n*, %)	0	0	Ns
Cycle length (day),mean ± SD	29.1 ± 3.0	28.6 ± 3.9	Ns
Progesterone levels (ng/mL)	20.1 ± 7.5	19.9 ± 9.4	Ns

## Supplementary Materials

The following are available online at https://www.mdpi.com/2077-0383/9/8/2634/s1. Figure S1. Western blot analysis of TM expression, Figure S2. Western blot analysis of ezrin expression.
