# Supplementary material for: Expression of Pinopodes in the Endometrium from Recurrent Pregnancy Loss Women. Role of Thrombomodulin and Ezrin
Source: J Clin Med. 2020 Aug 13;9(8):2634. doi: 10.3390/jcm9082634 (PMC7464296; doi:10.3390/jcm9082634)
Supplement: Supplementary file 1 [file jcm-09-02634-s001.pdf]

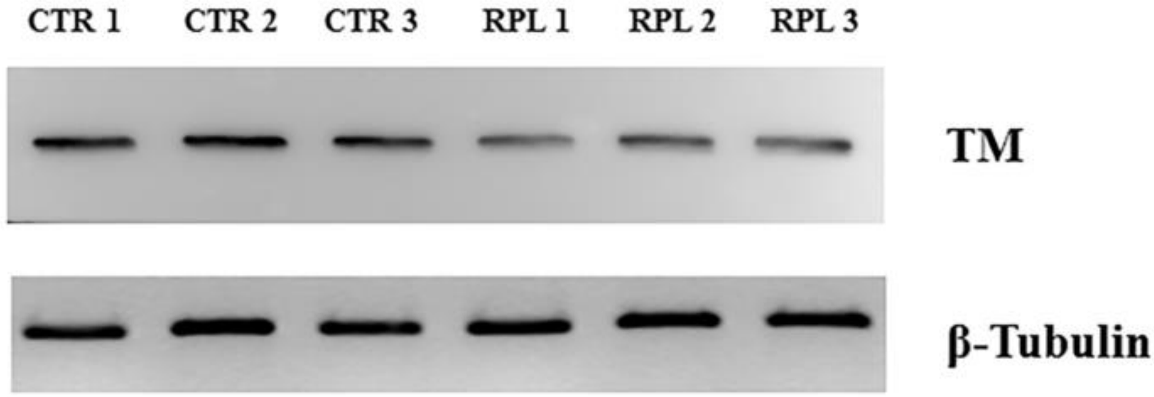

Figure S1. Western blot analysis. Full length gels obtained from RPL and CTR women for the analysis of TM. TM: thrombomodulin; CTR: control women; RPL: recurrent pregnancy loss women.

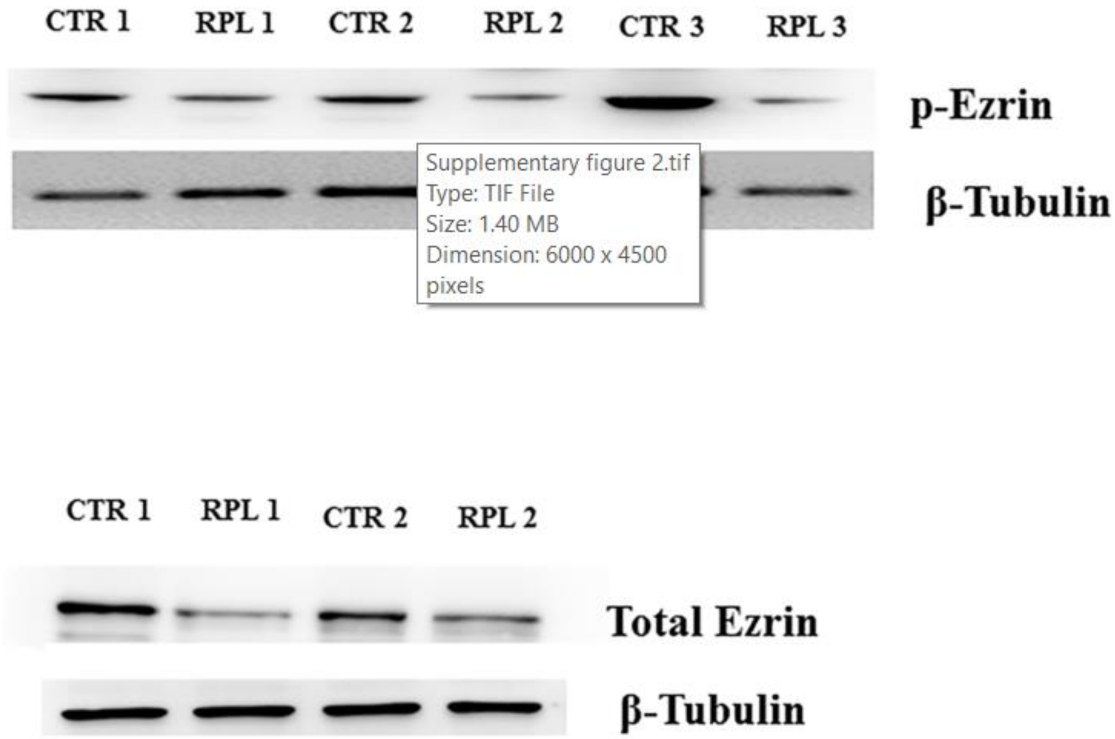

Figure S2. Western blot analysis. Full length gels obtained from RPL and CTR women for the analysis of phosphorilated and total ezrin. *p*-ezrin: phosphorilated ezrin; CTR: control women; RPL: recurrent pregnancy loss women.
